# The Etiology of Cancer

**Published:** 1891-01-31

**Authors:** 


### THE ETIOLOGY OF CANCER.

For many years pathologists have been searching for some definite cause to which may be assigned the various forms of cancer, and it has occurred to several workers that perhaps this disease might be traced to some minute organism. As long ago as 1854 Dr. Jabez Hogg wrote, "That terrible disease, cancer, will no doubt ultimately prove of vegetable growth, a conversion of the nutritive animal cell into that of the fungoid vegetable cell." In 1888 Professor Neisser published the results of some microscopical researches in which he described certain round bodies, which he considered vegetable spores, in cancerous tissue. Other observers have from time to time found microbes or spores in the cells of parts infected with this disease. On December 2nd, 1890, Dr. William Russell read a paper before the Pathological Society of London, in which he described fully and gave illustrations of certain small bodies which he had found in numerous sections of cancer. These minute structures stain readily with Fuchsin, and may be seen with a power of 100 diameters. They occur in groups of from two to five or more, with a clear space round them; they vary in size, some being larger than a red corpuscle of the blood. Their method of taking stains, their size, arrangement, and general appearance point to their vegetable nature. Dr. Russell thinks they belong to the sprouting fungi. If these organisms may be looked upon as the real cause of cancer, we should expect to find the disease to be contagious. Langenbeck is said to have produced cancer in animals by inoculation of the juice, but the evidence is quite as strong against as for this view, and Mr. Walter Sibley pointed out at the Pathological Society that the local infection of a neighbouring part (as the upper from the lower lip in cases of epithelioma) rarely took place. The whole question is as important a one as can be imagined, and further investigations must be waited for. It may be that these vegetable spores grow in the warm moisture of the cancer as in a congenial soil, without being more than accidental guests; that they are not to be looked upon as the "fons et origo mali," but merely as its concomitants.

